# Exclusive breastfeeding: Relation to gestational age, birth weight, and early neonatal ward admission. A nationwide cohort study of children born after 35 weeks of gestation

**DOI:** 10.1371/journal.pone.0285476

**Published:** 2023-05-24

**Authors:** Freja Marie Nejsum, Ragnhild Måstrup, Christian Torp-Pedersen, Ellen Christine Leth Løkkegaard, Rikke Wiingreen, Bo Mølholm Hansen

**Affiliations:** 1 Department of Pediatrics, Copenhagen University Hospital - North Zealand, Hillerød, Denmark; 2 Department of Neonatology, Copenhagen University Hospital - Rigshospitalet, Copenhagen, Denmark; 3 Department of Cardiology, Copenhagen University Hospital - North Zealand, Hillerød, Denmark; 4 Department of Public Health, University of Copenhagen, Copenhagen, Denmark; 5 Department of Gynaecology and Obstetrics, Copenhagen University Hospital - North Zealand, Hillerød, Denmark; 6 Department of Clinical Medicine, University of Copenhagen, Copenhagen, Denmark; American University of Beirut Medical Center, LEBANON

## Abstract

**Objectives:**

Prematurity, being small for gestational age and early neonatal ward admission are the major neonatal conditions that may interfere with breastfeeding supportive practices in infants born at gestational age ≥35+0 weeks. We aimed to investigate the associations between gestational age, small for gestational age, early neonatal ward admission and exclusive breastfeeding at one and four months.

**Methods:**

A register-based cohort-study of all Danish singletons with gestational age ≥35+0 weeks born in 2014–2015. In Denmark, health visitors routinely conduct free home visits throughout infants’ first year and thereby report breastfeeding data to The Danish National Child Health Register. These data were linked with data from other national registers. Logistic regression models estimated the odds ratio for exclusive breastfeeding at one and four months, adjusted for confounding variables.

**Results:**

The study population comprised 106,670 infants. Compared to gestational age 40 weeks, the adjusted odds ratio for exclusive breastfeeding at one month showed a decreasing tendency from gestational age ≥42 (n = 2,282) (1.07; 95% confidence interval (CI) 0.97–1.17) to 36 weeks (n = 2,062) (0.80; 95% CI 0.73–0.88). Small for gestational age (n = 2,342) was associated with decreased adjusted odds ratio for exclusive breastfeeding at one month (0.84; 95% CI 0.77–0.92). Neonatal ward admission was associated with increased adjusted odds ratio for exclusive breastfeeding at one month among late preterm infants (gestational age 35–36 weeks; n = 3,139) (1.31; 95% CI 1.12–1.54), as opposed to among early term (gestational age 37–38 weeks; n = 19,171) (0.84; 95% CI 0.77–0.92) and term infants (gestational age >38 weeks; n = 84,360) (0.89; 95% CI 0.83–0.94). The associations seemed to persist at four months.

**Conclusions:**

Decreasing gestational age and small for gestational age were associated with decreased exclusive breastfeeding rates. Neonatal ward admission was associated with increased exclusive breastfeeding rates among late preterm infants, whereas the opposite was observed among early term and term infants.

## Introduction

Breastfeeding provides important health benefits for both mothers and infants [1, 2], and The World Health Organization (WHO) recommends exclusive breastfeeding for the first six months after birth [3]. Factors in both mother and infant may interfere with the establishment of breastfeeding and it is important to identify conditions that predispose to breastfeeding difficulties to remedy complications by timely support.

In Denmark, 97% of all infants are born in the hospital [4] and approximately 70% are admitted to stay in the hospital after delivery for median two days [5, 6]. Maternity outpatient wards continue to offer breastfeeding support after hospital discharge until this task is undertaken by health visitors approximately one week postpartum. Health visitors conduct free home visits throughout the infant’s first year and the first home visit focuses on supporting breastfeeding establishment if the mother intends to breastfeed her baby.

There are well described prenatal factors that influence breastfeeding such as maternal body mass index, maternal age, socioeconomic status, smoking, parity, and delivery mode [7–9]. It is, however, possible to promote breastfeeding after delivery [10] and the Baby-friendly Hospital Initiative, launched by WHO and The United Nations Children’s Fund (UNICEF), has shown to improve breastfeeding rates [11]. In several cases, medical conditions may interfere with recommended key clinical practices, e.g., immediate and uninterrupted skin-to-skin contact, early initiation of breastfeeding, and no provision of any food or fluids to the breastfed infant [12]. In Denmark, infants with a gestational age (GA) < 35 weeks and 0 days are admitted to the neonatal ward after delivery because of prematurity, while healthy infants with GA ≥ 35 weeks and 0 days are admitted to the maternity ward where breastfeeding establishment is supported. It is recommended to routinely start early feeding with formula alongside breastfeeding establishment to prevent hypoglycemia in the first days of life if the infants are born preterm (GA < 37 weeks and 0 days) or small for gestational age (SGA) regardless of whether the infant is in the maternity ward or the neonatal ward. In Danish neonatal wards early feeding is generally given by cup or tube. Bottle is only used when exclusive breastfeeding is given up by the mother [13]. In Danish maternity wards cup is also preferred over bottle. Altogether approximately 10% of infants with a GA ≥ 35 and 0 days weeks are admitted to the neonatal ward after delivery because they need medical treatment [4] and, as in other countries, respiratory symptoms are the most common cause for admission [14, 15]. In approximately half of the Danish neonatal wards, infants and mothers can be admitted together directly from the delivery ward. In the rest of the Danish neonatal wards, mothers can sleep in the same room as their baby once they are discharged from the maternity ward. Thus, in infants born with a GA ≥ 35 weeks and 0 days preterm birth, being SGA, and early admission to the neonatal ward for medical treatment are the major conditions that may interrupt the establishment of breastfeeding. There is, however, a gap of knowledge on the breastfeeding rates in these infants.

Preterm birth has traditionally been considered a dichotomy, but increasing evidence suggests that, even in infants classified as term, GA should be considered a biological spectrum ranging from less to more mature [16]. Correspondingly, a systematic review suggests that breastfeeding rates decrease across the range of GAs from 41 to 37 weeks [17]. SGA refers to birth weights below the sex-specific expected birth weight according to GA. Two European studies suggest that SGA infants are less likely to establish breastfeeding compared to non-SGA infants [18, 19]. Studies from the United States have reported contradictory results on the association between neonatal ward admission and breastfeeding; some studies suggest that neonatal ward admission is associated with increased breastfeeding rates, while other studies suggest the opposite [20–23]. In one study the association depended on GA as late preterm infants admitted to the neonatal ward were more likely to breastfeed than late preterm infants admitted to the maternity ward, whereas the opposite was the case among term infants [20]. We aimed to investigate the influence of GA, SGA and early neonatal ward admission on exclusive breastfeeding at one and four months among infants with GA ≥ 35 weeks and 0 days by examining the associations between; 1) GA and exclusive breastfeeding at one and four months, 2) SGA and exclusive breastfeeding at one and four months, and 3) neonatal ward admission within 48 hours of birth and exclusive breastfeeding at one and four months stratified by GA divided in late preterm (GA 35 weeks and 0 days to 36 weeks and 6 days), early term (GA 37 weeks and 0 days to 38 weeks and 6 days), and term infants (GA > 38 weeks and 6 days). We hypothesized that low GA, SGA, and neonatal ward admission were associated with decreased exclusive breastfeeding rates.

## Methods

### Study design

We conducted a nationwide register-based cohort study, employing data from national registers held by Statistics Denmark and The Danish Health Data Authority. Data were unambiguously linked via unique Central Personal Register numbers that are assigned to all individuals with permanent residence in Denmark upon birth or immigration, and thus enable consistent linkage of data across registers [24]. The Danish Civil Registration System has previously been characterized, including strengths and limitations for register-based research [24, 25].

### Study population

The study population comprised all records of live-born singletons with GA ≥ 35 weeks and 0 days in The Danish Medical Birth Register between January 1, 2014, and December 31, 2015. We excluded infants with missing information on GA or birth weight, infants with GA or birth weight outliers, and infants where the mother or the infant died or migrated within four months of birth. Outliers were excluded under assumption of coding errors. GA outliers were defined as GA > 44 weeks and 6 days, and birth weight outliers were defined as birth weight beyond five standard deviations from the study population’s reference mean [26].

### Outcome variables

We investigated the two outcome variables ‘exclusive breastfeeding at one month’ and ‘exclusive breastfeeding at four months’ after delivery. Exclusive breastfeeding was defined in accordance with WHO´s definition [27] adapted to Danish conditions [28] as nourishing infants solely with breast milk besides water and maximum one formula feeding per week after hospital discharge. Exclusive breastfeeding at one month was considered an indicator of successful establishment of exclusive breastfeeding, persisting after hospital discharge. The Danish Health Authority recommends exclusive breastfeeding for six months, but it is also recommended to introduce complementary foods between four and six months after birth [28], and therefore we considered exclusive breastfeeding at four months an indicator of successful duration of exclusive breastfeeding.

Data on exclusive breastfeeding were retrieved from The Danish National Child Health Register administered by the Danish Health Data Authority. The Danish Health Authority recommends that all families in Denmark are offered minimum five free home visits by health visitors in the infant’s first year of life [29]. Danish parents are entitled to 52 weeks of paid parental leave and more than 95% utilize the health visitor services [30]. Health visitors routinely obtain data on exclusive breastfeeding cessation dates. It has been mandatory to report these data to The Danish National Child Health Register since 2011 [31]. Infants, who do not initiate exclusive breastfeeding, have no cessation date and thus no record on exclusive breastfeeding in The Danish National Child Health Register. In the present study, infants without a record on exclusive breastfeeding in The Danish National Child Health Register were therefore defined as not exclusive breastfeeding at one and four months.

### Covariates

We investigated three explanatory variables ‘GA’, ‘SGA’ and ‘neonatal ward admission’.

Data on GA and SGA were retrieved from The Danish Medical Birth Register. In Denmark, GA is routinely determined by first trimester ultrasonography, conducted in 92% of pregnancies [32]. GA was defined as completed pregnancy weeks and modelled as a categorical variable. It was divided into gestational weeks from + 0 to + 6 days and included 35, 36, 37, 38, 39, 40, 41, and ≥ 42 weeks (42 weeks and 0 days to 44 weeks and 6 days).

SGA was defined as birth weights two standard deviations below the study population’s reference mean sex-specific expected birth weight according to GA as described by Marsál et al [26]. Data on neonatal ward admission were retrieved from The Danish National Patient Register. Neonatal ward admission was defined as inpatient admissions to the pediatric specialty within 48 hours of birth. This time frame was appointed to eliminate transfers to the neonatal ward consequential to breastfeeding problems.

Factors in the registers, known to influence breastfeeding, were included as confounding variables; maternal smoking, maternal pre-pregnancy body mass index, maternal age at childbirth, maternal education, parity, delivery mode, and infant sex [7–9, 33]. Maternal education was considered an indicator of socioeconomic status and defined as the mother’s highest attained level of education upon parturition. If the mother solely had a record on highest attained educational level subsequently to parturition, the first available record was applied. The maternal educational levels were grouped into four categories: Level one (lowest) (International Standard Classification of Education 2011 (ISCED) 1–2), level two (ISCED 3), level three (ISCED 5–6), and level four (highest) (ISCED 7–8). Delivery mode was divided into vaginal delivery and caesarean section which was further subdivided into emergency and elective caesarean sections. Birthplace was included as a confounding variable to address residual confounding and divided in five groups (Region A-E), corresponding to the five health care regions of Denmark. The confounding variables were retrieved from The Danish Medical Birth Register, The Danish National Patient Register, and The Danish Education Registers. In Denmark, the Baby-friendly Hospital Initiative was stopped in 2008, thus no Danish hospitals hold a valid Baby-friendly Hospital Initiative designation, which was therefore not included as a confounding variable.

### Statistical analyses

We conducted three pairs of univariate and multivariate logistic regression analyses to examine the associations between:

1. GA and exclusive breastfeeding at one and four months. GA 40 weeks was used as reference. The multivariate analyses were adjusted for previously defined confounding variables and SGA.
2. SGA and exclusive breastfeeding at one and four months. The multivariate analyses were adjusted for previously defined confounding variables and GA.
3. Neonatal ward admission and exclusive breastfeeding at one and four months. The analyses were stratified by GA divided into late preterm, early term, and term infants. The multivariate analyses were adjusted for previously defined confounding variables and SGA.

It is a recognized problem that interpretation of odds ratios are nonintuitive, especially when the outcome of interest is common [34]. To facilitate interpretation of the odds ratios, we applied the multivariate logistic regression models to predict a standardized mother’s probability of exclusive breastfeeding at one and four months for each of the explanatory variables. The standardized mother was assigned the most frequent characteristics in the study population; smoking = no, body mass index = 18.5–24.0, age = 21–30 years, education = level two, parity = multiparous, delivery mode = vaginal delivery, infant’s sex = boy.

For each of the associations, we conducted two sets of sensitivity analyses to evaluate potential uncertainty associated with exclusive breastfeeding at one and four months: First, analyses employing a multivariate logistic regression model with misclassification correction as described by Liu et al [35]. Second, univariate and multivariate logistic regression analyses limited to infants with exclusive breastfeeding cessation dates in The Danish National Child Health Register, i.e., those who initiated exclusive breastfeeding.

All analyses were performed using R version 4.0.3. P-values ≤ 0.05 were considered statistically significant.

### Ethics

Danish law does not require ethical approval for register-based studies conducted for scientific research. The study was, however, approved by the data responsible institute in conformation with the General Data Protection Regulation (Capital Region of Denmark—Approval number: P-2019-280).

## Results

**Fig 1:**
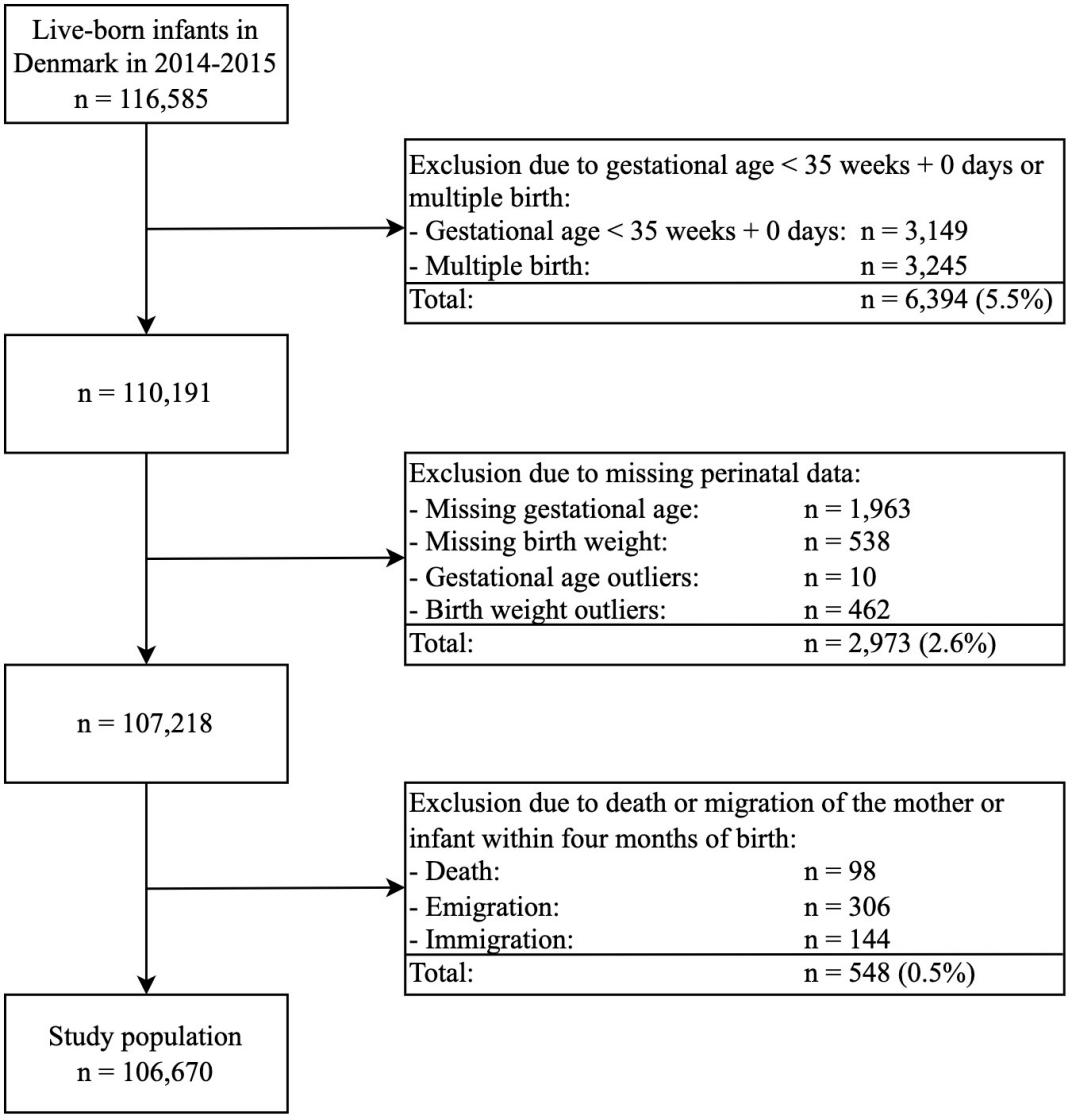
The study population.

The Medical Birth Register comprised 116,585 records on live-born infants from January 1, 2014, to December 31, 2015. We excluded 6,394 (5.5%) infants due to GA < 35 weeks and 0 days or multiple birth, 2,973 (2.6%) infants due to missing neonatal data, and 548 (0.5%) infants due to death or migration before follow-up. The study population included 106,670 infants, corresponding to 91.5% of the Danish birth cohort in the two-year period (Fig 1).

Table 1 shows baseline characteristics of the study population. Univariate and multivariate logistic regression analyses on the confounding variables’ association with exclusive breastfeeding at one and four months are shown in S1 Table. As expected, maternal smoking, high maternal body mass index, low maternal educational level, and caesarean section were strongly associated with decreased odds ratio for exclusive breastfeeding.

**Table 1:** Baseline characteristics of the study population.

Characteristic	Total, n (%)	Exclusive breastfeeding at one month			Exclusive breastfeeding at four months		
		Yes, n (%)	No, n (%)	p-value	Yes, n (%)	No, n (%)	p-value
	n = 106,670	n = 60,021	n = 46,649		n = 40,769	n = 65,901	
**Maternal smoking**							
Yes	11,757 (11.0%)	5,383 (9.0%)	6,374 (13.7%)		2,711 (6.7%)	9,046 (13.7%)	
Missing	786 (0.7%)	438 (0.7%)	348 (0.7%)	<0.001	271 (0.7%)	515 (0.8%)	<0.001
**Maternal body mass index**							
<18.5	4,873 (4.6%)	2,829 (4.7%)	2,044 (4.4%)		1,916 (4.7%)	2,957 (4.5%)	
18.5–24.9	65,663 (61.6%)	39,247 (65.4%)	26,416 (56.6%)		27,648 (67.8%)	38,015 (57.7%)	
25.0–29.9	22,048 (20.7%)	11,600 (19.3%)	10,448 (22.4%)		7,489 (18.4%)	14,559 (22.1%)	
≥30	13,294 (12.5%)	5,919 (9.9%)	7,375 (15.8%)		3,461 (8.5%)	9,833 (14.9%)	
Missing	792 (0.7%)	426 (0.7%)	366 (0.8%)	<0.001	255 (0.6%)	537 (0.8%)	<0.001
**Maternal age**							
≤20 years	2,239 (2.1%)	1,004 (1.7%)	1,235 (2.6%)		429 (1.1%)	1,810 (2.7%)	
21–30 years	53,810 (50.4%)	30,022 (50.0%)	23,788 (51.0%)		19,216 (47.1%)	34,594 (52.5%)	
31–40 years	48,282 (45.3%)	27,776 (46.3%)	20,506 (44.0%)		20,238 (49.6%)	28,044 (42.6%)	
≥41 years	2,339 (2.2%)	1,219 (2.0%)	1,120 (2.4%)	<0.001	886 (2.2%)	1,453 (2.2%)	<0.001
**Maternal education** ^1^							
Level one (lowest)	16,657 (15.6%)	7,620 (12.7%)	9,033 (19.4%)		4,037 (9.9%)	12,620 (19.1%)	
Level two	35,075 (32.9%)	18,671 (31.1%)	16,406 (35.2%)		11,216 (27.5%)	23,859 (36.2%)	
Level three	34,758 (32.6%)	20,756 (34.6%)	14,000 (30.0%)		15,141 (37.1%)	19,617 (29.8%)	
Level four (highest)	18,569 (17.4%)	12,111 (20.2%)	6,458 (13.8%)		9,758 (23.9%)	8,811 (13.4%)	
Missing	1,611 (1.5%)	860 (1.4%)	751 (1.6%)	<0.001	617 (1.5%)	991 (1.5%)	<0.001
**Birthplace**							
Region A	39,045 (36.6%)	25,261 (42.1%)	13,784 (29.5%)		18,666 (45.8%)	20,379 (30.9%)	
Region B	11,895 (11.2%)	6,368 (10.6%)	5,527 (11.8%)		3,920 (9.6%)	7,975 (12.1%)	
Region C	20,631 (19.3%)	9,851 (16.4%)	10,780 (23.1%)		6,066 (14.9%)	14,565 (22.1%)	
Region D	25,755 (24.1%)	14,660 (24.4%)	11,095 (23.8%)		9,628 (23.6%)	16,127 (24.5%)	
Region E	9,344 (8.8%)	3,881 (6.5%)	5,463 (11.7%)	<0.001	2,489 (6.1%)	6,855 (10.4%)	<0.001
**Parity**							
Primiparous	49,991 (46.9%)	28,699 (47.8%)	21,291 (45.6%)	<0.001	18,907 (46.4%)	31,084 (47.2%)	0.01
**Delivery mode**							
Emergency cesarean section	11,525 (10.8%)	5,874 (9.8%)	5,651 (12.1%)		3,769 (9.2%)	7,756 (11.7%)	
Elective cesarean section	9,513 (8.9%)	4,594 (7.7%)	4,919 (10.5%)		2,999 (7.3%)	6,514 (9.9%)	
Missing	104 (0.1%)	59 (0.1%)	45 (0.1%)	<0.001	42 (0.1%)	62 (1%)	<0.001
**Sex**							
Male	54,693 (51.3%)	30,577 (50.9%)	24,116 (51.7%)	0.01	20,472 (50.2%)	34,221 (51.9%)	<0.001

^1^Maternal education: Level one = International Standard Classification of Education 2011 (ISCED) 1–2, level two = ISCED 3, level three = ISCED 5–6, level four = ISCED 7–8.

Late preterm infants were predominantly admitted to the neonatal ward for more than five days with admitting diagnoses related to prematurity and low birth weight. In contrast, early term and term infants were predominantly admitted to the neonatal ward for less than five days with admitting diagnoses related to respiratory disorders (S2 Table).

Table 2 presents the unadjusted and adjusted odds ratio for exclusive breastfeeding at one and four months for each of the explanatory variables ‘GA’, ‘SGA’, and ‘neonatal ward admission’.

**Table 2:** Univariate and multivariate logistic regression analyses on the association between the explanatory variables and exclusive breastfeeding at one and four months.

Characteristic	Exclusive breastfeeding at one month			Exclusive breastfeeding at four months		
	Crude rates, n (%) n = 106,670	Unadjusted odds ratio (95% CI) n = 106,670	Adjusted odds ratio (95% CI) n = 103,564^1^	Crude rates, n (%) n = 106,670	Unadjusted odds ratio (95% CI) n = 106,670	Adjusted odds ratio (95% CI) n = 103,564^1^
**Gestational age** ^1^						
35 weeks (n = 1,077; 1.0%)	597 (55.4%)	0.91 (0.80–1.02)	1.04 (0.91–1.18)	383 (35.6%)	0.84* (0.74–0.95)	1.00 (0.88–1.14)
36 weeks (n = 2,062; 1.9%)	1,014 (49.2%)	0.71** (0.65–0.77)	0.80** (0.73–0.88)	666 (32.3%)	0.72** (0.66–0.80)	0.84** (0.76–0.93)
37 weeks (n = 4,795; 4.5%)	2,457 (51.3%)	0.77** (0.72–0.81)	0.87** (0.82–0.93)	1,584 (33.0%)	0.75** (0.70–0.80)	0.88** (0.82–0.94)
38 weeks (n = 14,376; 13.5%)	7,563 (52.6%)	0.81** (0.78–0.84)	0.91** (0.87–0.95)	4,956 (34.5%)	0.80** (0.77–0.83)	0.91** (0.87–0.96)
39 weeks (n = 24,217; 22.7%)	13,598 (56.1%)	0.93** (0.90–0.96)	0.98 (0.95–1.02)	9,213 (38.0%)	0.93** (0.90–0.96)	0.99 (0.95–1.02)
40 weeks (n = 32,045; 30.0%)	18,542 (57.9%)	1	1	12,733 (39.7%)	1	1
41 weeks (n = 25,816; 24.2%)	14,905 (57.7%)	1.00 (0.96–1.03)	1.03 (0.99–1.06)	10,264 (39.8%)	1.00 (0.97–1.04)	1.03 (0.99–1.06)
≥42 weeks (n = 2,282; 2.1%)	1,345 (58.9%)	1.05 (0.96–1.14)	1.07 (0.97–1.17)	970 (42.5%)	1.12* (1.03–1.22)	1.17* (1.06–1.28)
**Small for gestational age** ^2^						
Yes (n = 2,342; 2.2%)	1,173 (50.1%)	0.77** (0.71–0.84)	0.84** (0.77–0.92)	744 (31.7%)	0.75** (0.68–0.82)	0.84** (0.77–0.93)
No (n = 104,328; 97.8%)	58,848 (56.4%)	1	1	40,025 (38.4%)	1	1
**Neonatal ward admission (late preterm infants)** ^1^						
Yes (n = 1,307; 41.7%)	681 (52.1%)	1.05 (0.91–1.21)	1.31* (1.12–1.54)	405 (31.0%)	0.83* (0.71–0.96)	1.06 (0.89–1.26)
No (n = 1,832; 58.3%)	930 (50.8%)	1	1	644 (35.1%)	1	1
**Neonatal ward admission (early term infants)** ^1^						
Yes (n = 2,370; 12.4%)	1,063 (44.8%)	0.71** (0.65–0.78)	0.84** (0.77–0.92)	653 (27.6%)	0.70** (0.64–0.78)	0.84* (0.76–0.93)
No (n = 16,801; 87.6%)	8,957 (53.3%)	1	1	5,887 (35.0%)	1	1
**Neonatal ward admission (term infants)** ^1^						
Yes (n = 5,021; 6.0%)	2,629 (52.3%)	0.81** (0.76–0.85)	0.89** (0.83–0.94)	1,673 (33.3%)	0.76** (0.71–0.81)	0.87** (0.81–0.92)
No (n = 79,339; 94.0%)	45,761 (57.7%)	1	1	31,507 (39.7%)	1	1

Late preterm infants: Gestational age 35 weeks and 0 days to 36 weeks and 6 days. Early term infants: Gestational age 37 weeks and 0 days to 38 weeks and 6 days. Term infants: Gestational age > 38 weeks and 6 days.

*p-value<0.05,

**p-value<0.001

^1^The multivariate analyses were adjusted for maternal smoking, maternal pre-pregnancy body mass index, maternal age, maternal educational level, birthplace, parity, delivery mode, sex, and small for gestational age. Only complete cases were included in the multivariate analyses.

^2^The multivariate analyses were adjusted for maternal smoking, maternal pre-pregnancy body mass index, maternal age, maternal educational level, birthplace, parity, delivery mode, sex, and gestational age. Only complete cases were included in the multivariate analyses.

**Fig 2:**
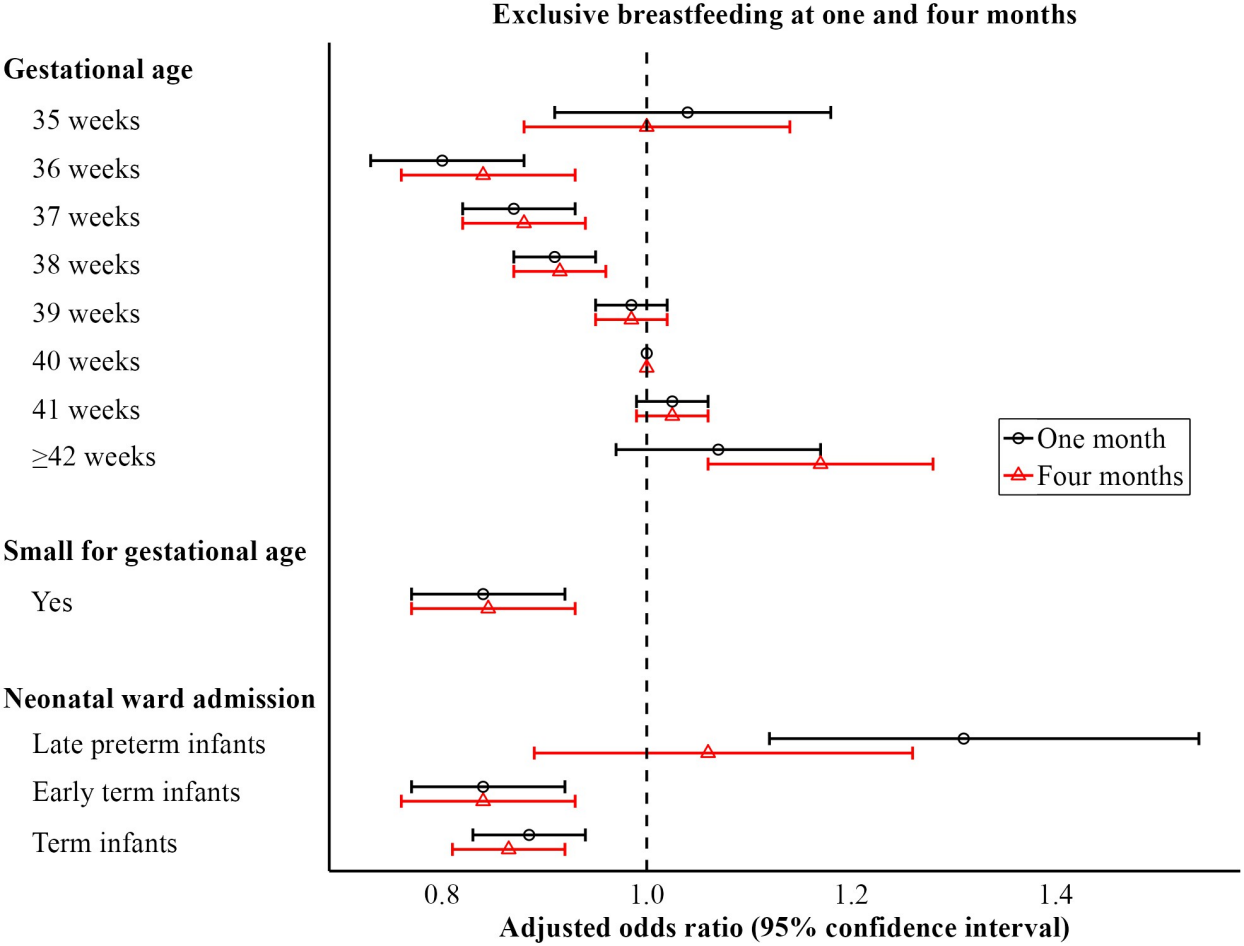
Adjusted odds ratio for exclusive breastfeeding at one and four months. Adjusted for maternal smoking, maternal pre-pregnancy body mass index, maternal age, maternal education, birthplace, parity, delivery mode, sex, and gestational age/small for gestational age.

The association between GA and exclusive breastfeeding at one month showed a decreasing tendency across the range of GAs from ≥ 42 to 36 weeks in both the univariate and multivariate analyses. Compared to GA 40 weeks, the adjusted odds ratio decreased from 1.07 (95% confidence interval (CI) 0.97–1.17) at GA ≥ 42 weeks to 0.80 (95% CI 0.73–0.88) at GA 36 weeks. The tendency deviated at GA 35 weeks where the adjusted odds ratio was 1.04 (95% CI 0.91–1.18) (Fig 2). The standardized mother’s probability of exclusive breastfeeding at one month was 59.6% (95% CI 58.4%-60.7%) for GA ≥ 42 weeks, 52.5% (95% CI 51.3%-53.8%) for GA 36 weeks, and 59.2% (95% CI 57.6%-60.8%) for GA 35 weeks (Table 3).

**Table 3:** The standardized mother’s probability of exclusive breastfeeding at one and four months.

Characteristic	Probability of exclusive breastfeeding at one month, % (95% CI)	Probability of exclusive breastfeeding at four months, % (95% CI)
**Gestational age** ^1^		
35 weeks	59.2 (57.6–60.8)	36.9 (35.3–38.5)
36 weeks	52.5 (51.3–53.8)	32.7 (31.5–33.8)
37 weeks	55.1 (54.3–56.0)	33.7 (32.9–34.5)
38 weeks	56.5 (55.9–57.1)	34.9 (34.4–35.5)
39 weeks	58.1 (57.6–58.6)	36.7 (36.2–37.2)
40 weeks	58.4 (58.0–58.9)	37.0 (36.5–37.5)
41 weeks	58.4 (57.9–58.9)	36.9 (36.4–37.4)
≥42 weeks	59.6 (58.4–60.7)	39.9 (38.7–41.0)
**Small for gestational age** ^2^		
Yes	53.8 (52.6–55.0)	32.8 (31.7–34.0)
No	58.5 (58.0–58.9)	37.0 (36.5–37.5)
**Neonatal ward admission (late preterm infant)** ^1^		
Yes	58.0 (55.1–60.9)	30.6 (27.8–33.2)
No	52.3 (49.6–55.1)	30.6 (28.0–33.1)
**Neonatal ward admission (early term infant)** ^1^		
Yes	52.7 (51.2–54.2)	31.7 (30.3–33.1)
No	57.2 (56.3–58.3)	35.5 (34.5–36.5)
**Neonatal ward admission (term infant)** ^1^		
Yes	54.7 (53.8–55.6)	33.0 (32.2–33.9)
No	58.4 (57.9–58.8)	37.1 (36.6–37.5)

Late preterm infants: Gestational age 35 weeks and 0 days to 36 weeks and 6 days. Early term infants: Gestational age 37 weeks and 0 days to 38 weeks and 6 days. Term infants: Gestational age > 38 weeks and 6 days.

^1^ Characteristics: Smoking = no, body mass index = 18.5–24.0, age = 21–30 years, education = level two, parity = multiparous, delivery mode = vaginal delivery, infant’s sex = boy, small-for-gestational-age = no.

^2^ Characteristics: Smoking = no, body mass index = 18.5–24.0, age = 21–30 years, education = level two, parity = multiparous, delivery mode = vaginal delivery, infant’s sex = boy, gestational age = 40 weeks and 0 days to 40 weeks and 6 days.

SGA was associated with decreased odds ratio for exclusive breastfeeding at one month in both the univariate and multivariate analyses. The adjusted odds ratio was 0.84 (95% CI 0.77–0.92) (Fig 2). The standardized mother’s probability of exclusive breastfeeding at one month was 58.5% (95% CI 58.0%-58.9%) for non-SGA infants and 53.8% (95% CI 52.6%-55.0%) for SGA infants (Table 3).

Neonatal ward admission was associated with increased odds ratio for exclusive breastfeeding at one month among late preterm infants, as opposed to among early term and term infants in both the univariate and multivariate analyses. The adjusted odds ratio was 1.31 (95% CI 1.12–1.54) in the late preterm group, 0.84 (95% CI 0.77–0.92) in the early term group, and 0.89 (95% CI 0.83–0.94) in the term group (Fig 2). The standardized mother’s probability of exclusive breastfeeding at one month, depending on neonatal ward admission versus no neonatal ward admission, was 58.0% (95% CI 55.1%-60.9%) versus 52.3% (95% CI 49.6%-55.1%) for late preterm infants, 52.7% (95% CI 51.2%-54.2%) versus 57.2% (95% CI 56.3%-58.3%) for early term infants and 54.7% (95% CI 53.8%-55.6%) versus 58.4% (95% CI 57.9%-58.8%) for term infants (Table 3).

The association between neonatal ward admission and exclusive breastfeeding was not statistically significant at four months among late preterm infants (adjusted odds ratio 1.06; 95% CI 0.89–1.26). The rest of the associations were substantially unchanged at four months.

The associations persisted in the sensitivity analyses employing the multivariate logistic regression model with misclassification correction (S3 Table). The associations between SGA and exclusive breastfeeding and neonatal ward admission of early term and term infants and exclusive breastfeeding disappeared however in the sensitivity analyses limited to infants with a record on exclusive breastfeeding in The Danish National Child Health Register (S4 Table).

## Discussion

In this nationwide register-based cohort study of infants born at GA ≥ 35 weeks and 0 days, we investigated major neonatal conditions expected to interfere with successful breastfeeding. We found a decreasing tendency in the probability of exclusive breastfeeding at one and four months across the range of GAs from ≥ 42 to 36 weeks. Admission to the neonatal ward seemed to promote breastfeeding in late preterm infants, whereas lower breastfeeding rates were observed in early term and term infants admitted to neonatal ward as well as in infants born SGA.

The study’s register-based design invokes several strengths. The nationwide study population and reliable accounting for censoring via Central Personal Registration numbers minimize selection bias. Furthermore, data on exclusive breastfeeding are generally susceptible to self-reporting and recall bias [36, 37], which in the present study were minimized by usage of health visitors’ routinely collected data on exclusive breastfeeding.

The main limitation of the study is potential misclassification of exclusive breastfeeding at one and four months. Infants, without a record on exclusive breastfeeding in The Danish National Child Health Register, were defined as not exclusive breastfeeding, but missingness could potentially further be due to rejection of the health visitor services or errors in reporting to The Danish National Child Health Register. However, more than 95% of Danish parents utilize the health visitor services [30], although minor deviations from the recommended five visits in the infant’s first year cannot be excluded. The exclusive breastfeeding rates were 56% at one month and 38% at four months. Similar breastfeeding rates have been found in a Danish study using weekly short message service text messages to obtain data on exclusive breastfeeding [38], but other Danish studies have reported markedly higher exclusive breastfeeding rates [39, 40]. Selection bias and differences in data collection are problematic for evaluation and comparison of breastfeeding rates which also was demonstrated by Flaherman et al investigating exclusive breastfeeding rates in California [36]. In the present study, known risk factors for early breastfeeding cessation were associated with decreased exclusive breastfeeding rates, suggesting internal validity. Potential residual confounding is another limitation of the study. We did not adjust for maternal self-efficacy or breastfeeding intention in the multivariate analyses as these data were unavailable [41], however nearly all Danish mothers intend to breastfeed [28, 42]. Furthermore, we did not adjust for ethnicity in the multivariate analyses as the available data were unsuitable to depict the complex association between ethnicity and breastfeeding. We included birthplace in the analyses to address residual confounding and we speculate that the observed variation in exclusive breastfeeding rates across birthplace also is influenced by the local approach and emphasis on breastfeeding.

Similar with previous findings, the present study found that maternal smoking, maternal BMI, maternal age, maternal education, parity, delivery mode and sex influence exclusive breastfeeding rates [7–9]. Our study furthermore suggests that GA, SGA, and early neonatal ward admission influence exclusive breastfeeding rates, although the effect sizes of these factors seem to be smaller than the effect sizes of the well described prenatal factors.

Previous studies suggest that both late preterm [43] and early term birth [44] are risk factors for not exclusively breastfeeding after hospital discharge. A systematic review has further reported a dose-effect like association between GA and breastfeeding initiation and continuation, but none of the included studies examined exclusive breastfeeding after hospital discharge. Correspondingly, a systematic review suggests that breastfeeding rates decrease across the range of GAs from 41 to 37 weeks [17]. Our study suggests that the dose-effect like association also applies to exclusive breastfeeding after hospital discharge and thereby supports the accumulating evidence that GA represents a biological spectrum from less to more mature, even in term infants [16].

The admission criteria and clinical practices in maternity and neonatal wards vary globally [45]. Despite these variations, the guidelines for preventing neonatal hypoglycemia seem to be consistent. These recommend preventing neonatal hypoglycemia in late preterm infants and infants born SGA by frequent feeding in the first day of life [46], and early introduction of formula as supplementation to breastfeeding if needed is a widespread practice in these infants [47]. Although the observational design of our study did not provide us with further data to investigate putative causes, we believe our data suggest that late preterm infants benefit from the approaches to breastfeeding support in the neonatal ward, as we consider it unlikely that those admitted to the neonatal ward had advantageous physiological conditions for breastfeeding. The positive association between neonatal ward admission and exclusive breastfeeding did, however, seem to weaken at four months, indicating that this group may need continuous support to maintain exclusive breastfeeding. This lends support to previous findings that suggest a trend towards higher breastfeeding initiation rates and lower breastfeeding continuation rates among late preterm infants admitted to the neonatal ward compared to late preterm infants admitted to the maternity ward, although not statistically significant [21, 22].

Neonatal ward admission was contrastingly associated with decreased exclusive breastfeeding rates among early term and term infants. Early term and term infants were generally admitted to the neonatal ward for a short time and breastfeeding may consequentially not have been well-established before discharge from the neonatal ward. The association indicates that support, provided in the short time spent in the neonatal ward, did not fully compensate for barriers to breastfeeding within these groups. These findings are consistent with those of other studies examining the association between neonatal ward admission and breastfeeding among term infants and support Colaizy et al who reported that the association between neonatal ward admission and breastfeeding depends on GA [20, 23].

SGA was associated with decreased exclusive breastfeeding rates. This is consistent with previous studies [18, 19], but the underlying causes are not properly understood. Dooks et al have speculated that in utero events predispose SGA infants to compromised breastfeeding effectivity [19]. Furthermore, Danish guidelines to prevent neonatal hypoglycemia recommend early feeding with formula in the first day after birth in SGA infants, which also may impede breastfeeding [48]. Another potentially contributing factor is that parents and medical professionals closely monitor SGA infants’ weight and may be inclined to give supplemental formula feeding to ensure catch-up weight gain even though it is recommended in Denmark that SGA infants are exclusively breastfed for the first six months of life.

Breastfeeding establishment in the hospital is influenced by factors in infants, mothers, and clinical practices [42, 49] intertwined in a complex interaction. Underlying drivers for variations in breastfeeding rates are thus likely to be intercorrelated. In our study the associations between the major conditions, expected to interfere with breastfeeding, were substantially unchanged from one to four months postpartum, except for the association between neonatal ward admission and exclusive breastfeeding among late preterm infants. This could indicate that GA, SGA, and neonatal ward admission of early term and term infants mainly are risk factors for not establishing exclusive breastfeeding. Future studies should target interventions to promote the establishment of breastfeeding in infants born SGA, late preterm infants admitted to the maternity ward and early term and term infants admitted to the neonatal ward.

## Conclusions

We found that decreasing GA from ≥ 42 to 36 weeks and being SGA were associated with decreased probability of exclusive breastfeeding. Neonatal ward admission was associated with increased probability of exclusive breastfeeding among late preterm infants, as opposed to among early term and term infants. This indicates that late preterm infants seem to benefit from approaches to breastfeeding support in the neonatal ward.

## Supporting information

S1 Table Univariate and multivariate logistic regression analyses on the confounding variables’ association to exclusive breastfeeding at one and four months. (PDF)

S2 Table Characteristics of the neonatal ward admissions. (PDF)

S3 Table Sensitivity analyses: Multivariate logistic regression model with misclassification correction. (PDF)

S4 Table Sensitivity analyses: Infants with a record on exclusive breastfeeding in The Danish National Child Health Register. (PDF)
