# Supplementary material for: Exclusive breastfeeding: Relation to gestational age, birth weight, and early neonatal ward admission. A nationwide cohort study of children born after 35 weeks of gestation
Source: PLoS One. 2023 May 24;18(5):e0285476. doi: 10.1371/journal.pone.0285476 (PMC10208505; doi:10.1371/journal.pone.0285476)
Supplement: S1 Table — (PDF) [file pone.0285476.s001.pdf]

S1 Table. Univariate and multivariate logistic regression analyses on the confounding variables' association to exclusive breastfeeding at one and four months.

Characteristic	Exclusive breastfeeding at one month		Exclusive breastfeeding at four months	
	Unadjusted odds ratio (95% CI) n=106,670	Adjusted odds ratio[1] (95% CI) n=103,564	Unadjusted odds ratio (95% CI) n=106,670	Adjusted odds ratio[1] (95% CI) n=103,564
**Maternal smoking**				
Yes (n=11,757; 11.0%)	0.62** (0.60-0.65)	0.75** (0.72-0.78)	0.45** (0.43-0.47)	0.61** (0.58-0.64)
No (n=94,127; 88.3%)	1	1	1	1
**Maternal body mass index**				
<18.5 (n=4,873; 4.6%)	0.93* (0.88-0.99)	0.99 (0.93-1.06)	0.89** (0.84-0.94)	0.99 (0.93-1.06)
18.5 - 24.9 (n=65,663; 61.6%)	1	1	1	1
25.0 - 29.9 (n=22,048; 20.7%)	0.75** (0.72-0.77)	0.84** (0.81-0.87)	0.71** (0.68-0.73)	0.82** (0.79-0.85)
≥30 (n=13,294; 12.5%)	0.54** (0.52-0.56)	0.67** (0.64-0.69)	0.48** (0.46-0.51)	0.63** (0.61-0.66)
**Maternal age**				
≤20 years (n=2,239; 2.1%)	0.60** (0.55-0.65)	0.94 (0.86-1.04)	0.33** (0.30-0.37)	0.69** (0.61-0.77)
21 - 30 years (n=53,810; 50.4%)	0.93** (0.91-0.95)	1.07** (1.04-1.10)	0.77** (0.75-0.79)	0.97* (0.94-1.00)
31 - 40 years (n=48,282; 45.3%)	1	1	1	1
≥41 years (n=2,339; 2.2%)	0.80** (0.74-0.87)	0.85** (0.78-0.93)	0.84** (0.78-0.92)	0.91* (0.83-0.99)
**Maternal education[2]**				
Level one (lowest) (n=16,657; 15.6%)	0.45** (0.43-0.47)	0.59** (0.56-0.62)	0.29** (0.28-0.30)	0.43** (0.41-0.45)
Level two (n=35,075; 32.9%)	0.61** (0.59-0.63)	0.76** (0.73-0.79)	0.42** (0.41-0.44)	0.56** (0.54-0.59)
Level three (n=34,758; 32.6%)	0.79** (0.76-0.82)	0.90** (0.87-0.94)	0.70** (0.67-0.72)	0.82** (0.79-0.85)
Level four (highest) (n=18,569; 17.4%)	1	1	1	1
**Birthplace**				
Region A (n=39,045; 36.6%)	1	1	1	1
Region B (n=11,895; 11.1%)	0.63** (0.60-0.66)	0.72** (0.69-0.75)	0.54** (0.51-0.56)	0.67** (0.64-0.70)
Region C (n=20,631; 19.3%)	0.50** (0.48-0.52)	0.54** (0.52-0.56)	0.45** (0.44-0.47)	0.53** (0.51-0.55)
Region D (n=25,755; 24.1%)	0.72** (0.70-0.74)	0.74** (0.72-0.77)	0.65** (0.63-0.67)	0.70** (0.67-0.72)
Region E (n=9,344; 8.8%)	0.39** (0.37-0.41)	0.41** (0.39-0.43)	0.40** (0.38-0.42)	0.44** (0.42-0.47)
**Parity**				
Primiparous (n=49,991; 46.9%)	1.09** (1.06-1.12)	1.04* (1.01-1.07)	0.97* (0.94-0.99)	0.95** (0.92-0.97)
Multiparous (n=56,679; 53.1%)	1	1	1	1
**Delivery mode**				
Emergency caesarean section (n=11,525; 10.8%)	0.76** (0.73-0.79)	0.82** (0.79-0.86)	0.74** (0.71-0.77)	0.82** (0.78-0.85)
Elective caesarean section (n=9,513; 8.9%)	0.68** (0.65-0.71)	0.71** (0.68-0.74)	0.70** (0.67-0.73)	0.71** (0.67-0.75)
Vaginal delivery (n=85,528; 80.2%)	1	1	1	1
**Sex**				
Male (n=54,693; 51.3%)	0.97* (0.95-0.99)	0.97* (0.95-1.00)	0.93** (0.91-0.96)	0.95** (0.92-0.97)
Female (n=51,977; 48.7%)	1	1	1	1

*p-value<0.05, **p-value<0.001

[1]Adjusted for maternal smoking, maternal pre-pregnancy body mass index, maternal age, maternal educational level, birthplace, parity, delivery mode, sex, gestational age, and being small for gestational age. Only complete cases were included in the multivariate models.

[2]Maternal education: Level one = International Standard Classification of Education 2011 (ISCED) 1-2, level two = ISCED 3, level three = ISCED 5-6, level four = ISCED 7-8.
