# Supplementary material for: Exclusive breastfeeding: Relation to gestational age, birth weight, and early neonatal ward admission. A nationwide cohort study of children born after 35 weeks of gestation
Source: PLoS One. 2023 May 24;18(5):e0285476. doi: 10.1371/journal.pone.0285476 (PMC10208505; doi:10.1371/journal.pone.0285476)
Supplement: S2 Table — (PDF) [file pone.0285476.s002.pdf]

S2 Table. Characteristics of the neonatal ward admissions

	**Late preterm infants** n=1,307	**Early term infants** n=2,370	**Term infants** n=5,021
**Admitting diagnosis**			
Neurology	23 (1.8%)	178 (7.5%)	863 (17.2%)
Respiratory	130 (9.9%)	765 (32.3%)	1,819 (36.2%)
Infection	7 (0.5%)	45 (1.9%)	541 (10.8%)
Hypoglycemia	28 (2.1%)	172 (7.3%)	241 (4.9%)
Prematurity or low birth weight	979 (74.9%)	255 (10.8%)	112 (2.2%)
Other	140 (10.7%)	955 (40.3%)	1,439 (28.7%)
**Length of stay**			
Less than two days	214 (16.4%)	1,058 (44.6%)	2,149 (42.8%)
Two to five days	200 (15.3%)	641 (27.0%)	1,628 (32.4%)
More than five days	893 (68.3%)	671 (28.3%)	244 (24.8%)

Late preterm infants: Gestational age 35-36 weeks. Early term infants: Gestational age 37-38 weeks. Term infants: Gestational age > 38 weeks.
