# Supplementary material for: Exclusive breastfeeding: Relation to gestational age, birth weight, and early neonatal ward admission. A nationwide cohort study of children born after 35 weeks of gestation
Source: PLoS One. 2023 May 24;18(5):e0285476. doi: 10.1371/journal.pone.0285476 (PMC10208505; doi:10.1371/journal.pone.0285476)
Supplement: S3 Table — (PDF) [file pone.0285476.s003.pdf]

S3 Table. Sensitivity analyses: Multivariate logistic regression model with misclassification correction

Characteristic	Exclusive breastfeeding at one month<br>Adjusted odds ratio (95% CI)<br>n=103,564	Exclusive breastfeeding at four months<br>Adjusted odds ratio (95% CI)<br>n=103,564
**Gestational age[1]**		
35 weeks (n=1,077; 1.0%)	1.03 (0.93-1.13)	1.00 (0.90-1.10)
36 weeks (n=2,062; 1.9%)	0.84** (0.76-0.92)	0.85** (0.72-0.93)
37 weeks (n=4,795; 4.5%)	0.90** (0.84-0.96)	0.87** (0.81-0.93)
38 weeks (n=14,376; 13.5%)	0.93** (0.91-0.95)	0.91** (0.89-0.93)
39 weeks (n=24,217; 22.7%)	0.99 (0.97-1.01)	0.98 (0.96-1.00)
40 weeks (n=32,045; 30.0%)	1	1
41 weeks (n=25,816; 24.2%)	1.00 (0.98-1.02)	1.00 (0.98-1.02)
≥42 weeks (n=2,282; 2.1%)	0.99 (0.92-1.06)	1.06 (0.99-1.13)
**Small for gestational age[2]**		
Yes (n=2,342; 2.2%)	0.86** (0.79-0.93)	0.84** (0.78-0.93)
No (n=104,328; 97.8%)	1	1
**Neonatal ward admission (late preterm infants)[1]**		
Yes (n=1,255; 41.4%)	1.41* (1.18-1.64)	1.04 (0.81-1.23)
No (n=1,779; 58.6%)	1	1
**Neonatal ward admission (early term infants)[1]**		
Yes (n=2,271; 12.3%)	0.86* (0.78-0.94)	0.86* (0.78-0.94)
No (n=16,253; 87.7%)	1	1
**Neonatal ward admission (term infants)[1]**		
Yes (n=4,835; 5.9%)	0.92** (0.88-0.96)	0.88** (0.83-0.93)
No (n=77,171; 94.1%)	1	1

Late preterm infants: Gestational age 35-36 weeks. Early term infants: Gestational age 37-38 weeks. Term infants: Gestational age > 38 weeks.
*p-value<0.05, **p-value<0.001
[1]The multivariate analyses were adjusted for maternal smoking, maternal pre-pregnancy body mass index, maternal age, maternal educational level, birthplace, parity, delivery mode, sex, and small for gestational age. Only complete cases were included in the multivariate analyses.
[2]The multivariate analyses were adjusted for maternal smoking, maternal pre-pregnancy body mass index, maternal age, maternal educational level, birthplace, parity, delivery mode, sex, and gestational age. Only complete cases were included in the multivariate analyses.
