# Supplementary material for: Exclusive breastfeeding: Relation to gestational age, birth weight, and early neonatal ward admission. A nationwide cohort study of children born after 35 weeks of gestation
Source: PLoS One. 2023 May 24;18(5):e0285476. doi: 10.1371/journal.pone.0285476 (PMC10208505; doi:10.1371/journal.pone.0285476)
Supplement: S4 Table — (PDF) [file pone.0285476.s004.pdf]

S4 Table. Sensitivity analyses: Infants with a record on exclusive breastfeeding in The Danish National Child Health Register

Characteristic	Exclusive breastfeeding at one month		Exclusive breastfeeding at four months	
	Unadjusted odds ratio (95% CI) n=76,086	Adjusted odds ratio (95% CI) n=73,953	Unadjusted odds ratio (95% CI) n=76,086	Adjusted odds ratio (95% CI) n=73,953
**Gestational age[1]**				
35 weeks (n=723; 1.0%)	1.18 (0.97-1.43)	1.41** (1.15-1.72)	0.92 (0.79-1.07)	1.11 (0.95-1.30)
36 weeks (n=1,353; 1.8%)	0.75** (0.66-0.85)	0.88 (0.77-1.00)	0.79** (0.71-0.89)	0.93 (0.83-1.05)
37 weeks (n=3,246; 4.3%)	0.78** (0.71-0.85)	0.94 (0.85-1.02)	0.78** (0.72-0.84)	0.91* (0.84-0.99)
38 weeks (n=9,979; 13.1%)	0.78** (0.74-0.82)	0.92* (0.87-0.98)	0.81** (0.77-0.85)	0.92** (0.87-0.97)
39 weeks (n=17,301; 22.7%)	0.91** (0.87-0.96)	0.98 (0.93-1.03)	0.93** (0.90-0.97)	0.99 (0.95-1.03)
40 weeks (n=23,152; 30.4%)	1	1	1	1
41 weeks (n=18,631; 24.5%)	0.99 (0.95-1.04)	1.04 (0.99-1.09)	1.00 (0.97-1.04)	1.03 (0.99-1.07)
≥42 weeks (n=1,701; 2.2%)	0.94 (0.83-1.06)	1.08 (0.95-1.23)	1.09 (0.98-1.20)	1.09 (0.98-1.21)
**Small for gestational age[2]**				
Yes (n=1,543; 2.0%)	0.84* (0.75-0.85)	0.95 (0.84-1.08)	0.80** (0.72-0.89)	0.92 (0.82-1.02)
No (n=74,543; 98.0%)	1	1	1	1
**Neonatal ward admission (late preterm infants)[1]**				
Yes (n=860; 41.4%)	1.17 (0.94-1.44)	1.53** (1.19-1.95)	0.79* (0.66-0.94)	0.96 (0.78-1.18)
No (n=1,216; 58.6%)	1	1	1	1
**Neonatal ward admission (early term infants)[1]**				
Yes (n=1,454; 11.0%)	0.85* (0.78-0.92)	1.03 (0.90-1.18)	0.79** (0.74-0.85)	0.95 (0.85-1.08)
No (n=11,771; 89.0%)	1	1	1	1
**Neonatal ward admission (term infants)[1]**				
Yes (n=3,413; 5.6%)	0.85** (0.76-0.97)	0.99 (0.91-1.08)	0.81** (0.73-0.91)	0.92* (0.85-0.99)
No (n=57,372; 94.4%)	1	1	1	1

Late preterm infants: Gestational age 35-36 weeks. Early term infants: Gestational age 37-38 weeks. Term infants: Gestational age > 38 weeks.
*p-value<0.05, **p-value<0.001
[1]The multivariate analyses were adjusted for maternal smoking, maternal pre-pregnancy body mass index, maternal age, maternal educational level, birthplace, parity, delivery mode, sex, and small for gestational age. Only complete cases were included in the multivariate analyses.
[2]The multivariate analyses were adjusted for maternal smoking, maternal pre-pregnancy body mass index, maternal age, maternal educational level, birthplace, parity, delivery mode, sex, and gestational age. Only complete cases were included in the multivariate analyses.
